# Dimensionless Numbers to Analyze Expansive Growth Processes

**DOI:** 10.3390/plants8010017

**Published:** 2019-01-10

**Authors:** Joseph K. E. Ortega

**Affiliations:** Department of Mechanical Engineering, University of Colorado Denver, Denver, CO 80217-3364, USA; joseph.ortega@ucdenver.edu

**Keywords:** dimensionless numbers, Π parameters, expansive growth, biophysical equations, water uptake, wall deformation, *Chara corallina*, *Phycomyces blakesleeanus*, *Pisum satinis* L.

## Abstract

Cells of algae, fungi, and plants have walls and exhibit expansive growth which can increase their volume by as much as 10,000 times. Expansive growth is central to their morphogenesis, development, and sensory responses to environmental stimuli. Equations describing the biophysical processes of the water uptake rate and the wall deformation rate have been derived, validated, and established. A significant amount of research provides insight into the molecular underpinnings of these processes. What is less well known are the relative magnitudes of these processes and how they compare during expansive growth and with walled cells from other species. Here, dimensionless numbers (Π parameters) are used to determine the magnitudes of the biophysical processes involved in the expansive growth rate of cells from algae (*Chara corallina*), fungi (*Phycomyces blakesleeanus*), and plants (*Pisum satinis* L.). It is found for all three species that the cell’s capability for the water uptake rate is larger than the wall plastic deformation rate and much larger than the wall elastic deformation rate. Also, the wall plastic deformation rates of all three species are of similar magnitude as their expansive growth rate even though the stress relaxation rates of their walls are very different. It is envisioned that dimensionless numbers can assist in determining how these biophysical processes change during development, morphogenesis, sensory responses, environmental stress, climate change, and after genetic modification.

## 1. Introduction

Both water uptake and cell wall deformation are required for the expansive growth of walled cells. Expansive growth may be understood as a sequence of biophysical processes. Initially, active solutes inside the plasma membrane create an osmotic pressure difference across the plasma membrane and water flows into the cell. The increase in cell volume requires stretching (deforming) the cell wall. Stress inside the cell wall is generated as the pressure inside the plasma membrane (*P*_i_) increases to values higher than that outside (*P*_o_), producing turgor pressure (*P* = *P*_i_ − *P*_o_). Both cell wall stress and turgor pressure increase as water uptake continues. When *P* equals Δπ, water is prevented from flowing into the cell. If the wall’s deformation to the wall stress is elastic (reversible wall deformation), then the magnitude of the wall stress and deformation will increase and decrease as *P* increases and decreases. Thus, the increase in wall deformation and the resulting increase in volume are not permanent, and this does not represent expansive growth. However, it shows that the water flow into the cell is regulated by changing *P*, and in turn depends on elastic deformation and the elastic properties of the wall.

Permanent wall deformation (plastic deformation) is achieved when biochemical reactions inside the cell wall break the load-bearing bonds between the wall polymers and reduce the wall stress, producing wall stress relaxation. Wall stress relaxation is accompanied by turgor pressure relaxation and the smaller *P* produces water flow into the cell, causing plastic and elastic deformation of the wall. Cell wall polymers are continuously added to the inner surface of the wall to prevent thinning and rupture. As water enters the cell, elastic deformation of the cell wall chamber continues simultaneous to the plastic deformation until both the wall stress and *P* achieve the magnitude that moves water into the cell at the same volumetric rate as the wall volumetric plastic deformation rate, resulting in an equilibrium *P*. The plastic deformation of the wall produces a cell wall chamber that is permanently larger. Now the cell wall chamber and the volume it encloses are permanently larger, this is considered expansive growth. 

During normal growth, the biochemical reactions that loosen the wall, wall stress and pressure relaxation, water uptake, and subsequent plastic and elastic deformation of the cell wall chamber occur simultaneously and continuously [1]. However, the description of the sequential individual events highlights the important events and the importance of the elastic deformation of the wall that is needed to produce *P*. The magnitude of *P* is then used to regulate the water flow rate into the cell that produces the permanent wall deformation after the stress relaxation. During constant stress relaxation and plastic deformation rate of the wall (steady expansive growth rate), *P* is maintained by the elastic wall deformation (and a non-zero Δπ) at a smaller magnitude (turgor pressure depression) that will produce a constant water uptake rate that matches the stress relaxation rate and the plastic deformation rate of the cell wall. 

### 1.1. Biophysical Equations

Interdependent biophysical equations have been derived, validated, and established that describe the interrelated biophysical processes of the net water uptake rate, the total wall deformation rate, and the rate of change of the turgor pressure; see [2] and the references within. Equation (1) describes in relative terms, the rate of change of the water volume in the cell, *v*_w_, as the difference in the volumetric rate of the water uptake, *L* (Δπ − *P*), and transpiration, *v*_T_ (see Nomenclature for a description and definitions of individual variables and terms).

(1) $$v_{w}=L\left(\Delta \pi -P\right)-v_{T}$$

Equation (2) describes in relative terms, the rate of change in the volume of the cell wall chamber, *v*_cw_, as the sum of the volumetric irreversible (plastic) deformation rate, *ϕ* (*P* − *P*_C_), and the volumetric reversible (elastic) deformation rate, (1/*ε*) d*P*/d*t*, of the cell wall.

(2) $$v_{\mathrm{cw}}=\phi \left(P-P_{C}\right)+\left(\frac{1}{\varepsilon }\right)\frac{dP}{dt}$$

Equation (3) describes the rate of change of the turgor pressure, *P*.

(3) $$\left(\frac{1}{\varepsilon }\right)\frac{dP}{dt}=L\left(\Delta \pi -P\right)-v_{T}-\phi \left(P-P_{C}\right)$$

### 1.2. Dimensionless Biophysical Equations

Dimensional analysis was conducted on Equations (1)–(3), producing Equations (4)–(6); the details of the dimensional analysis are presented in [3]. The variables in Equations (1)–(3) are made dimensionless (*) with the following constant reference parameters: *v*_s_ (steady or average relative volumetric growth rate), *v*_sT_ (steady or average relative volumetric transpiration rate) and *P*_C_ (critical turgor pressure); see [3] for details. The dimensionless variables are designated with an asterisk (*).

(4) $${v_{w}}^{*}=\Pi_{\mathrm{wv}}\left(\Delta \pi^{*}-P^{*}\right)-\Pi_{\mathrm{Tv}}\;{v_{T}}^{*}$$

(5) $${v_{\mathrm{cw}}}^{*}=\Pi_{\mathrm{pv}}\left(P^{*}-1\right)+\Pi_{\mathrm{ev}}\frac{dP^{*}}{dt^{*}}$$

(6) $$\frac{dP^{*}}{dt^{*}}=\Pi_{\mathrm{we}}\left(\Delta \pi^{*}-P^{*}\right)-\Pi_{\mathrm{Te}}{v_{T}}^{*}-\Pi_{\mathrm{pe}}\left(P^{*}-1\right)$$

It is important to note that because Equations (4)–(6) are dimensionless, the magnitude of the dimensionless coefficients (Π parameters) at the beginning of each term reflects the magnitude of that term [3]. If the reader is not familiar with dimensionless Π parameters and their application to plant, algal and fungal cells, please see the short review [4].

### 1.3. Dimensionless Π Parameters

The dimensionless Π parameters at the beginning of each term in Equations (4)–(6) correspond to the following Π parameters. The interpretation of the dimensionless Π parameters is expressed as ratios of biophysical processes [3].

$$\Pi_{\mathrm{wv}}=\left(\frac{L\;P_{C}}{v_{s}}\right)=\left(\frac{relative\;volumetric\;water\;uptake\;rate}{relative\;volumetric\;growth\;rate}\right)$$

$$\Pi_{\mathrm{Tv}}=\left(\frac{v_{\mathrm{sT}}}{v_{s}}\right)=\left(\frac{relative\;volumetric\;transpiration\;rate}{relative\;volumetric\;growth\;rate}\right)$$

$$\Pi_{\mathrm{pv}}=\left(\frac{\phi \;P_{C}}{v_{s}}\right)=\left(\frac{relative\;volumetric\;plastic\;deformation\;rate\;of\;the\;wall}{relative\;volumetric\;growth\;rate}\right)$$

$$\Pi_{\mathrm{ev}}=\left(\frac{P_{C}}{\varepsilon }\right)=\left(\frac{relative\;volumetric\;elastic\;deformation\;rate\;of\;the\;wall}{relative\;volumetric\;growth\;rate}\right)$$

$$\Pi_{\mathrm{we}}=\left(\frac{\varepsilon \;L}{v_{s}}\right)=\left(\frac{relative\;volumetric\;water\;uptake\;rate}{relative\;volumetric\;elastic\;deformation\;rate\;of\;the\;wall}\right)$$

$$\Pi_{\mathrm{Te}}=\left(\frac{\varepsilon \;v_{\mathrm{sT}}}{P_{C}\;v_{s}}\right)=\left(\frac{relative\;volumetric\;transpiration\;rate}{relative\;volumetric\;elastic\;deformation\;rate\;of\;the\;wall\;}\right)$$

$$\Pi_{\mathrm{pe}}=\left(\frac{\varepsilon \;\phi }{v_{s}}\right)=\left(\frac{relative\;volumetric\;plastic\;deformation\;rate\;of\;the\;wall}{relative\;volumetric\;elastic\;deformation\;rate\;of\;the\;wall}\right)$$

### 1.4. Overview of Present Research

Here, the magnitude of the dimensionless Π parameters, Π_wv_, Π_Tv_, Π_pv_, and Π_ev,_ is calculated for fungal, algal, and plant cells, and used to analyze the biophysical processes of the water uptake, transpiration, plastic and elastic wall deformations during expansive growth. It is found that during expansive growth, the magnitude of the water uptake rate (Π_wv_) exceeds those rates of the other biophysical processes (Π_Tv_, Π_pv_, and Π_ev_) by approximately ten times or more (order of magnitude or more). It is also found that the plastic deformation rate is between one and three orders of magnitude greater than the wall elastic deformation rate. The biophysical processes involved in regulating the rate of change of the turgor pressure are also analyzed. It is found that the turgor pressure time rate of change is dominated by the ratio of the net water uptake rate and the elastic deformation rate, i.e., Π_we_. The large values of Π_we_ indicate that relatively large rates of water uptake can be produced and sustained by the small elastic deformation rates of the cell wall. Also, it is found that another dimensionless Π parameter, Π_wd_, can determine the ratio of the *net* water uptake rate and the *total* wall deformation rate during expansive growth. It was found that the net water uptake rates exceed the total wall deformation rate by approximately one to two orders of magnitude. This finding reveals that the cell wall deformation rate limits and controls the expansive growth rate, and the biophysical equation that describes the wall deformation rate can be used to describe the expansive growth rate. It was previously shown that the rate of wall stress relaxation and turgor pressure relaxation are central to expansive growth [1,5] and a dimensionless governing equation was obtained and solved [3,5]. It was shown that the turgor pressure relaxation and stress relaxation rates are controlled by the magnitude of Π_pe_. It was found that Π_pe_ is large, one to three orders of magnitude greater than unity, and the magnitude is very different for the species of walled cells considered. Here, the components of Π_pe_ are further analyzed and it is learned that both Π_pv_ and Π_ev_ contribute to the variability of the magnitude of Π_pe_ for different species of walled cells. The implications of this finding are discussed.

## 2. Results

The magnitude of each Π parameter can be determined for each walled cell from the magnitude of the biophysical variables (*v*_s_, *v*_sT_, *L*, *P*_C_, *ε*, and *ϕ*) determined for that cell. Typically, the magnitudes of these biophysical variables are determined from in vivo creep and in vivo stress relaxation experiments conducted with the pressure probe; see Appendix A for more details and references.

### 2.1. Values of Variables from Fungal, Algal, and Plant Cells

The biophysical variables used in the dimensionless Π parameters are obtained from pressure probe studies conducted on growing sporangiophores of *P. blakesleeanus* [6,7], the internodes of *C. corallina* [8,9], and cells in the stem tissue of *P. satinis* L. [10], and summarized by Ortega [5]. Additional values for the hydraulic conductivity of the plasma membrane, *L*_p_, were obtained for *P. blakesleeanus* [11], *C. corallina* [12], and *P. satinis* L. [10]. The average values for these biophysical variables are shown in Table 1.

### 2.2. Values for Π Parameters from Fungal, Algal, and Plant Cells

Using the magnitudes of the biophysical variables in Table 1, the magnitude of the respective dimensionless parameters are calculated and summarized in Table 2.

**Figure 1:**
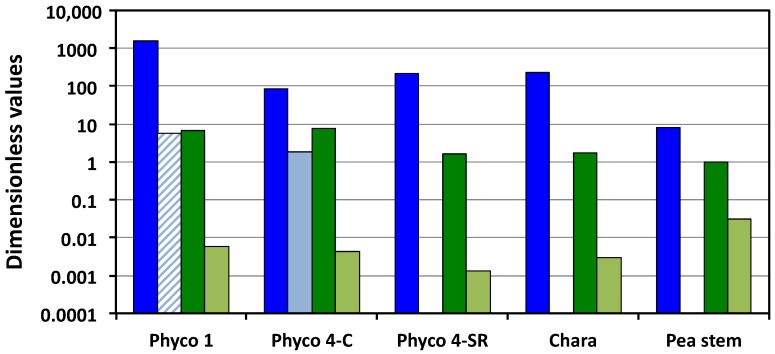
Magnitudes of Π_wv_ (dark blue), Π_Tv_ (light blue), Π_pv_ (dark green) and Π_ev_ (light green) for the cell species and types presented in Table 2. The striped light blue bar indicates that the magnitude uses an approximate value of *v*_sT_ for stage I. Note that the vertical scale is logarithmic.

The magnitudes of the Π parameters presented in Table 2 can be read in a couple of ways. Reading the values down each column allows the reader to view the magnitudes of the dimensionless coefficients in Equations (4)–(6) for each species or stage of the walled cell. Reading the magnitudes of the Π parameters across each row allows the reader to compare the values of the same Π parameter for different species or stages of the walled cell. For a visual comparison, the magnitudes of Π_wv_, Π_Tv_, Π_pv_, and Π_ev_ are plotted in Figure 1 for all cell species and types presented in Table 2.

**Figure 2:**
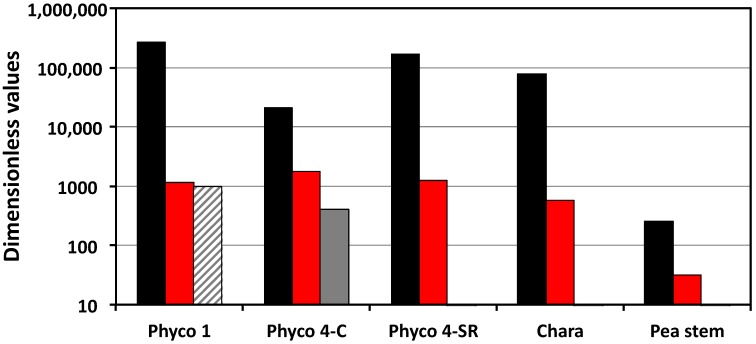
Magnitudes of Π_we_ (black), Π_pe_ (red), and Π_Te_ (gray) for the cell species and types presented in Table 2. The striped gray bar indicates that the magnitude uses an approximate value of *v*_sT_ for stage I. Note that the vertical scale is logarithmic.

The last three dimensionless coefficients in Table 2 (Π_we_, Π_Te_, and Π_pe_) determine the magnitude of the dimensionless rate of change of the turgor pressure, Equation (6), and are presented in Figure 2. The turgor pressure will change with changes in the rates of water uptake, transpiration, and expansive growth (stress relaxation). 

### 2.3. Comparing Net Water Uptake and Total Wall Deformation Rates

In a system of interdependent equations, sometimes it is useful to compare the magnitudes of entire equations because the comparison can provide information and insights, and lead to simplifications that allow accurate approximate solutions. The expansive growth rate of walled cells is described by two simultaneous and interrelated biophysical processes, and modeled by two interdependent biophysical equations, Equations (4) and (5). A comparison of the magnitudes of the *net* water uptake rate, Equation (4), to that of the *total* wall deformation rate, Equation (5), can be conducted using dimensionless Π parameters. Since the magnitude of each term in the dimensionless Equations (4) and (5) is the preceding dimensionless Π, simply summing the dimensionless Π coefficients gives the magnitude of each equation.

|Equation (4); the *net* water uptake rate| = Π_wv_ − Π_Tv_

|Equation (5); the *total* wall deformation rate| = Π_pv_ + Π_ev_

**Figure 3:**
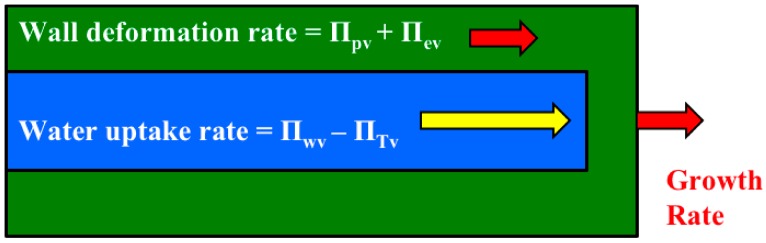
A schematic illustration of the cross-section of a cylindrical walled cell showing the simultaneous and interrelated biophysical processes of the *net* water uptake rate (yellow on blue) and the *total* wall deformation rate (red on green). It is shown that the expansive growth rate is limited by the smallest rate of the two biophysical processes, e.g., the wall deformation rate in this illustration.

In the case of the expansive growth rate of walled cells, the equation with the smallest magnitude governs the overall growth rate because the overall growth rate cannot exceed the smallest (limiting) rate of either the water uptake or the wall deformation (Figure 3). 

Another dimensionless Π parameter, Π_wd_, can be used to compare the magnitudes of Equations (4) and (5), i.e., Equation (7) [5].

(7) $$\Pi_{\mathrm{wd}}=\left(\frac{\Pi_{\mathrm{wv}}-\Pi_{\mathrm{Tv}}}{\Pi_{\mathrm{pv}}+\Pi_{\mathrm{ev}}}\right)=\frac{magnitude\;of\;net\;water\;uptake\;rate}{magnitude\;of\;wall\;deformation\;rate}$$

**Figure 4:**
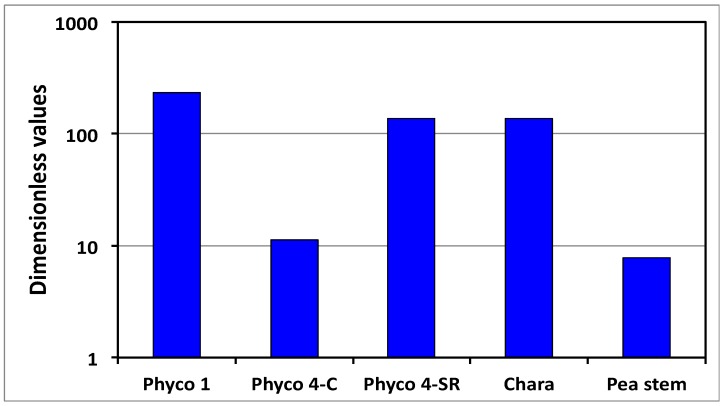
Magnitudes of Π_wd_ for the cell species and types presented in Table 2. Note that the vertical scale is logarithmic.

So, if the magnitude of Π_wd_ is greater than unity, the magnitude of the net water uptake rate is the largest and the wall deformation rate is the limiting process. Then, the limiting equation, Equation (5), can be used as the governing equation for the overall expansive growth rate. The magnitude of Π_wd_ is calculated for the walled cells presented in Table 2 (see Appendix B for calculations), and the results are presented in Figure 4. The results presented in Figure 4 show that the magnitude of Π_wd_ is much greater than unity for each of the walled cells in Table 2.

## 3. Discussion

### 3.1. Results

Here, the biophysical processes of the water uptake, transpiration, plastic and elastic wall deformations are analyzed for fungal, algal, and plant cells using dimensionless Π parameters. The Π parameters are obtained from dimensionless biophysical equations describing the net water uptake rate and the total cell wall deformation rate; Equations (4) and (5) respectively. The use of dimensionless Π parameters provides an assessment of the magnitude of each biophysical process during the expansive growth for each cell type considered. In Table 2, reading the values down each column, it can be seen that the magnitude of Π_wv_ is the largest dimensionless coefficient in Equations (4) and (5) for each species of walled cell (the first four values in each column). Figure 1 presents a visual comparison of the relevant Π parameters, i.e., Π_wv_, Π_Tv_, Π_pv_ and Π_ev_. Π_wv_ is the ratio of the relative volumetric water uptake rate and the relative volumetric growth rate, so the large magnitudes (greater than unity) indicate that the biophysical processes responsible for the water uptake are capable of transporting water into the cells at a much faster rate than they are growing. The magnitudes of Π_pv_ indicate that the cell walls are capable of relative volumetric plastic deformation rates that are equal to or slightly greater than the relative volumetric growth rate, but all values are of the same order of magnitude, between unity and 7.7. In contrast, the small magnitudes of Π_ev_ indicate that during expansive growth, the walls are capable of only small relative volumetric elastic deformation rates, much smaller than the relative volumetric growth rate; Π_ev_ << 1 (Figure 1). In the one case where transpiration rates were measured, *P. blakesleeanus* stage IV (C), it can be seen that the sporangiophores capability for the water uptake rate is much greater than that for water loss through transpiration, by more than an order of magnitude. It can be seen that for the growing cells of pea stems (*P. satinis* L.), the internodes of *C. corallina*, and the sporangiophores of *P. blakesleeanus* (stage I and stage IV), the magnitudes of the Π parameters representing the water uptake rates are larger than those representing the transpiration rates, the plastic wall deformation rates, and the elastic wall deformation rates by one to five orders of magnitude (Figure 1). This finding indicates that the rate of water uptake is the dominant process during the expansive growth of these cells. Also, it is found that the magnitudes of the plastic wall deformation rates are larger than the magnitudes of the elastic wall deformation rates by one to three orders of magnitude (Π_pv_ and Π_ev_ in Figure 1). 

The dimensionless rate of change of the turgor pressure is a function of Π_we_, Π_Te_, and Π_pe_ (Equation (6)). The results presented in Table 2 and Figure 2 show that the values of Π_we_ are larger than those of Π_Te_ and Π_pe_ by one to two orders of magnitude. It is expected that Π_we_ is large because Π_wv_ is generally large (Π_wv_ >> 1) and Π_ev_ is generally small (Π_ev_ << 1), and Π_we_ = Π_wv_/Π_ev_. The large magnitude of Π_we_ indicates that a high rate of water uptake can be achieved and sustained with a relatively small rate of elastic wall deformation. The magnitude of Π_Te_ was only determined for the intact stage IV sporangiophores, and its magnitude is nearly the same as that of Π_pe_. The results presented in Figure 2 indicate that the dimensionless rate of change of the turgor pressure is dominated by the magnitude of Π_we_ (black bars). During normal growth, changes in the transpiration rate and the expansive growth rate (stress relaxation rate) produce relatively small changes in the rate of change of turgor pressure and in the magnitude of equilibrium turgor pressure.

It is shown that the relative magnitudes of whole equations can be determined by simply summing the magnitudes of the Π parameters that precede the dimensionless term in each dimensionless equation (Figure 3). Another dimensionless number, Π_wd_, was obtained to determine the relative magnitudes of the *net* water uptake rate and the *total* wall deformation rate [5], see Equation (7). The magnitudes of Π_wd_ for the cell species and types presented in Table 2 are calculated (Appendix B) and presented in Figure 4. The results demonstrate that Π_wd_ is approximately one to two orders of magnitude greater than unity for all the cells in Table 2. Thus, it is concluded for these cells that the smaller wall deformation rate limits and governs the expansive growth rate. This conclusion indicates that either Equation (2) or Equation (5) may be used as the sole governing equation for the expansive growth rate for these cells in normal growing conditions. 

### 3.2. Stress Relaxation and the Π_pe_ Parameter

When Π_wd_ > 1, as shown in Figure 4, the expansive growth rate is limited and governed by the cell wall deformation rate; Equation (2) or Equation (5). Prior research demonstrates that the in vivo expansive growth rate requires stress relaxation of the cell wall in order to produce the decrease in turgor pressure that creates the water potential difference necessary to drive water uptake [1,5]. Stress relaxation experiments are conducted by eliminating the water uptake and transpiration for the growing walled cell and measuring the decreasing turgor pressure as a function of time using a pressure probe [6,10]. The dimensionless governing equation for the turgor pressure relaxation and the wall stress relaxation can be obtained from Equation (5) by recognizing that *v*_cw_* = *v*_w_* = 0 when both water uptake and transpiration rates are zero for these experimental conditions. Then, the dimensionless rate of change of the turgor pressure is reduced to Equation (8) [3,5].

(8) $$\frac{dP^{*}}{dt^{*}}=-\frac{\Pi_{\mathrm{pv}}}{\Pi_{\mathrm{ev}}}\left(P^{*}-1\right)=-\Pi_{\mathrm{pe}}\left(P^{*}-1\right)$$

Equation (8) is integrated to obtain a solution for the dimensionless turgor pressure as a function of dimensionless time, Equation (9) [3,5].

(9) $$P^{*}=\left(P_{i}^{*}-1\right)\exp\;(-\Pi_{\mathrm{pe}}t^{*})+1$$

Equation (9) demonstrates that the dimensionless turgor pressure, P*, decays exponentially from an initial value, *P*_i_*, to unity with a dimensionless time constant of *t*_c_* = (Π_pe_)^−1^. So, the dimensionless stress relaxation rate is determined by the magnitude of the dimensionless number, Π_pe_. The mean values of Π_pe_ are presented in Table 2 and plotted in Figure 2 (red bars). It is shown that the Π_pe_ values for the sporangiophores of *P. blakesleeanus* are an order of magnitude larger than those of the internode cells of *C. corallina* and two orders of magnitude larger than those of the cells in the stem tissue of *P. satinis* L. 

Previously, it was shown that if the magnitude of Π_pe_, *ϕ*, and *ε* are known, the steady expansive growth rate, *v*_s_, can be calculated with good accuracy [5]. These calculations demonstrate for the growing sporangiophores of *P. blakesleeanus* that a single constant value for Π_pe_ (Π_pe_ = 1524) can be used to determine the expansive growth rate of the sporangiophore during different developmental stages (stage I and stage IV) and different growth conditions (plucked from the mycelium and growing with its base in pure water) [5]. Similarly, for plant cells of the growing stem of *P. satinis* L., it was found that a single constant value for Π_pe_ (Π_pe_ = 32) can be used to determine the expansive growth rate of the stem after the application of the growth hormone IAA and during different growth conditions (incised and growing in water, and just cut from the plant) [5]. These findings suggest that the magnitude of Π_pe_ is a constant characteristic of the wall of each cell species and individual cell. The implications of this finding are significant because it demonstrates that the dimensionless steady and quasi-steady expansive growth rate of the walled cell is directly related to the magnitude of Π_pe_. Furthermore, this finding indicates that the ‘wall stress relaxation’ similarity can be achieved by matching the magnitude of Π_pe_ [5].

The large magnitude of Π_pe_ and the large differences in values obtained for the cells of *P. blakesleeanus*, *C. corallina*, and *P. satinis* L. were previously discussed [5]. A few observations are noted from this comparison of Π_pe_ values (Figure 2). (a) All the magnitudes of Π_pe_ for these cells are much greater than unity (Π_pe_ >> 1), indicating that the plastic deformation rate is much greater than the elastic deformation rate. This finding draws into question modeling the cell wall with constitutive equations that only describe the elastic wall deformation. (b) Mechanical energy is continually dissipated by the cell wall at a relatively large rate during the normal expansive growth and the cell walls of the sporangiophores of *P. blakesleeanus* dissipate mechanical energy at a significantly higher rate than the cell walls in the pea stems of *P. satinis* L. (c) The large differences in the magnitudes of Π_pe_ for the different cell species suggest that wall loosening chemistry may be qualitatively different for the fungal sporangiophores, algal internodes, and plant tissue. There is experimental evidence indicating that the molecular wall loosening mechanisms for plant and algal cells are different [14,15]. Little is known about wall loosening in the cell walls of the sporangiophores of *P. blakesleeanus* other than low pH can elicit creep [16]. However, because the molecular compositions of fungal cell walls are chitin-based [17] and different from those of the plants and algae (cellulose based), it is reasonable to think that the molecular agents for wall loosening might be different. 

The large differences in values of Π_pe_ obtained for cells of *P. blakesleeanus*, *C. corallina*, and *P. satinis* L. can be further analyzed using the new results presented here. Two dimensionless Π parameters, Π_pv_ and Π_ev_, contribute to the magnitude of Π_pe_; Π_pe_ = Π_pv_/Π_ev_. The results presented in Table 2 and Figure 1 reveal that all the values of Π_pv_ are relatively similar, i.e., of the same order of magnitude, varying between unity and 7.7. The values of Π_ev_ are slightly more variable, varying by over an order of magnitude. Overall, some of the differences in the values of Π_pe_ are because of the variability in the relative volumetric plastic deformation rate of the wall. However, the variability of the relative volumetric elastic deformation rate of the wall contributes equally (or slightly more) to the differences in the magnitude of Π_pe_ and the dimensionless stress relaxation rate of the different species of cells analyzed. 

Here, it is hypothesized that both the plastic and elastic wall deformation rates are regulated during the expansive growth. The variability of Π_pv_ and Π_ev_ presented in Table 2 and Figure 1 supports this hypothesis. Additional support is obtained from the algal internode cells of *C. corallina*, where it is found that the longitudinal volumetric elastic modulus of the wall decreased in magnitude (thus increasing the wall elastic deformation rate) as the elongation growth rate increased in magnitude [8]. For these internode algal cells, both the plastic and the elastic deformation rates of the wall increase as the elongation rate increases. It is generally thought that the plastic deformation rate of the wall is regulated at a microscopic level by breaking load-bearing bonds between wall polymers. Controlling the number of unbroken and load-bearing bonds between polymers, and making the wall polymers deform elastically when the wall is stressed, can regulate the elastic deformation rate. It is envisioned that the cell wall’s plastic and elastic deformation rates may be regulated at the microscopic level by controlling the rate of detachment of load-bearing bonds, *k*_d_, and controlling the rate of attachment, *k*_a_, and the formation of new bonds to become load-bearing [18]. Future research should determine whether the elastic deformation rate is regulated independently, or is a function of the plastic deformation rate regulation. It is noted that this finding draws into question the modeling of the cell wall with constitutive equations that only describe the plastic wall deformation.

### 3.3. Similarity Analysis

Similarity between qualitatively identical processes is achieved when the dimensionless Π parameters governing the processes are the same magnitude [19]. This similarity principle was recently employed on the results of constant tension creep experiments. Creep extension was observed for frozen–thawed sporangiophore walls when the pH of the bathing solution was decreased to acidic values [16]. Furthermore, it was found that the measured creep rates are within the range of the elongation growth rates observed from natural growing sporangiophores. The similarity principle was employed to determine whether this low pH mechanism (acid growth mechanism [20]) might be responsible for producing wall loosening at a molecular level, and therefore used to initiate, maintain and regulate the expansive growth of the sporangiophore wall. The experimental protocol was slightly modified to obtain the biophysical variables defining the Π_pe_ parameter [5]. Then, the magnitude of Π_pe_ was determined and compared to the values obtained during normal growth [5]. Wall stress relaxation similarity is achieved when the magnitudes of Π_pe_ for the two deforming walls (frozen–thawed walls and normal growing walls) are the same. If the Π_pe_ values are the same, it is evidence that regulating the pH of the cell wall is a viable mechanism for initiating, maintaining, and controlling wall loosening in the sporangiophore during normal growth. However, it was found that the magnitude of Π_pe_ was an order of magnitude smaller than that obtained during normal growth [5]. Therefore, it was concluded that lowering the pH may contribute to normal wall extension and regulation, but other agents must be involved to achieve the value of Π_pe_ observed for normal growing sporangiophore walls. Now, because of the new findings presented here, it is suggested that both the plastic and the elastic wall deformation rates, Π_pv_ and Π_ev_, be determined in future experiments to learn whether the respective rates obtained from the constant tension creep experiments are similar to those obtained from naturally growing sporangiophores. The results could help determine whether one of the wall deformation processes is similar to those obtained from naturally growing sporangiophores and the other is not, or if both wall deformation processes (plastic and elastic) are not similar.

Wall stress relaxation similarity was also employed to guide and validate a local numerical model of the cell wall. Recently, a statistical numerical model was constructed for cell wall extension using the same cell wall loosening mechanism employed by growing cells, i.e., breaking load-bearing bonds between cell wall polymers and making the bonds under zero-load conditions [18]. Two variables in the model are *k*_d_ and *k*_a_ (the rate of detachment of load-bearing bonds and the rate of making bonds under zero-load conditions, respectively). In this model, the plastic deformation of the wall is the result of breaking load-bearing bonds between microfibrils and connecting polymer tethers. The elastic deformation of the wall is the result of stretching the polymer tethers between microfibrils. It was found that the experimentally obtained stress relaxation curves for the fungal sporangiophores and plant tissue (even though very different) could be accurately modeled when the Π_pe_ values that were obtained experimentally for sporangiophores and plant tissue were used in the model [18]. Using the same Π_pe_ for each cell species establishes wall stress relaxation similarity between the biological cell wall and the statistical model of the cell wall. In addition, the elongation growth response to steps-up in the turgor pressure could be accurately described by the statistical model when the respective experimentally obtained values of Π_pe_ for the sporangiophores (*P. blakesleeanus*) and the algal cells (*C. corallina*) were used [18]. Interestingly, it was found that Π_pe_ is related to one of the microscopic variables in the statistical model, *k*_d_ (the detachment rate between microfibrils and connecting polymer tethers); Π_pe_ = *k*_d_/*v*_s_. Future research will explore how the relative magnitudes of *k*_d_ and *k*_a_ change during different growth responses produced by environmental sensory stimuli and steps-up and steps-down in the turgor pressure.

### 3.4. Additional Future Research

In the future, it is envisioned that quantitative analysis employing dimensionless Π parameters can assist in the determination of which biophysical processes are changed, and by how much, during development, morphogenesis, sensory responses, environmental stresses (water, temperature, and mineral stresses) and climate change. Furthermore, dimensionless parameters can assist in determining which biophysical processes are altered in growth mutants of the walled cells. Dimensionless Π parameters should be obtained for the additional variables needed to model the plant cells in tissue and organs [2], such as the pressure and the solute concentration in the apoplasm. It would seem that dimensionless Π parameters would be especially useful for honing crop plants to new environments and in the face of climate change. 

## Figures and Tables

**Table 1:** Biophysical variables determined for four different cell types; stage I and stage IV sporangiophores of *P. blakesleeanus*, the algal internode cells of *C. corallina*, and cells from the stem tissue of *P. satinis* L. The values obtained from in vivo creep and in vivo stress relaxation experiments are designated with C and SR, respectively. The transpiration rate is zero for the stage IV sporangiophores of *P. blakesleeanus* during stress relaxation experiments (stage 4-SR), the submerged algal internode cells of *C. corallina*, and the interior cells from the growing tissue of the stem of *P. satinis* L. Transpiration rates for the stage IV sporangiophores (stage 4-C) were measured [13], but those for stage I were not and because the stage I sporangiophores transpire; the values measured for stage IV sporangiophores were used as an approximation (identified with an asterisk).

	*P. blakesleeanus* Stage 1-C	*P. blakesleeanus* Stage 4-C	*P. blakesleeanus* Stage 4-SR	*C. corallina* Internode-C	*P. satinis* L. Stem-C, SR
*v*_s_ (h^−1^)	0.0210	0.0680	0.0083	0.0039	0.0751
*L* (h^−1^ MPa^−1^)	83	23	23	3	2
*P*_C_ (MPa)	0.40	0.26	0.08	0.30	0.30
*v*_sT_ (h^−1^)	0.12 *	0.12	0.0	0.0	0.0
*ε* (MPa)	68.9	60.9	60.9	100.0	9.5
*ϕ* (h^−1^ MPa^−1^)	0.350	2.000	0.169	0.022	0.250

**Table 2:** Magnitudes of Π parameters calculated using the magnitudes of the biophysical variables presented in Table 1. The asterisks adjacent to the numbers in the stage I column indicate that the dimensionless values were obtained with an approximate value for the steady or average relative volumetric transpiration rate (*v*_sT_) (see Table 1).

	*P. blakesleeanus* Stage I-C	*P. blakesleeanus* Stage IV-C	*P. blakesleeanus* Stage IV-SR	*C. corallina* Internode-C	*P. satinis* L. Stem-C, SR
$\Pi_{\mathrm{wv}}=\left(\frac{L\;P_{C}}{v_{s}}\right)$	1581	88	222	231	8
$\Pi_{\mathrm{Tv}}=\left(\frac{v_{\mathrm{sT}}}{v_{s}}\right)$	5.7 *	1.8	0.0	0.0	0.0
$\Pi_{\mathrm{pv}}=\left(\frac{\phi \;P_{C}}{v_{s}}\right)$	6.7	7.7	1.6	1.7	1.0
$\Pi_{\mathrm{ev}}=\left(\frac{P_{C}}{\varepsilon }\right)$	0.0058	0.0043	0.0013	0.0030	0.0316
$\Pi_{\mathrm{we}}=\left(\frac{\varepsilon \;L}{v_{s}}\right)$	272,319	20,599	168,759	76,923	253
$\Pi_{\mathrm{Te}}=\left(\frac{\varepsilon \;v_{\mathrm{sT}}\;}{P_{C}\;v_{s}}\right)$	984 *	413	0.0	0.0	0.0
$\Pi_{\mathrm{pe}}=\left(\frac{\varepsilon \;\phi }{v_{s}}\right)$	1148	1791	1240	564	32

## Nomenclature

A=area of the plasma membrane
$L_{P}=hydraulic\;conductivity\;of\;the\;plasma\;membrane$
$L=\left(\frac{L_{p\;}A}{V}\right)=relative\;hydraulic\;conductance\;of\;the\;plasma\;membrane$
P=turgor pressure (gage pressure) relative to the atmosphere
$P_{C}=critical\;turgor\;pressure\;\left(to\;be\;exceeded\;before\;plastic\;extension\;begins\right)$
t=time
V=volume
$V_{\mathrm{cw}}=volume\;of\;the\;cell\;wall\;chamber$
$V_{w}=volume\;of\;water\;in\;the\;cell$
$V_{T}=volume\;of\;water\;lost\;through\;transpiration$
$v=\left(\frac{dV}{V\;dt}\right)=relative\;rate\;of\;change\;in\;volume\;of\;the\;cell$
$v_{\mathrm{cw}}=\left(\frac{dV_{\mathrm{cw}}}{V_{\mathrm{cw}}\;dt}\right)=relative\;rate\;of\;change\;in\;volume\;of\;the\;cell\;wall\;chamber$
$v_{s}=\left(\frac{dV}{V\;dt}\right)=steady\;or\;quasi-steady\;relative\;rate\;of\;change\;in\;volume\;of\;the\;cell$
$v_{\mathrm{sT}}=\left(\frac{dV_{T}}{V_{T}\;dt}\right)=steady\;or\;quasi-steady\;v_{T}$
$v_{T}=\left(\frac{dV_{T}}{V_{T}\;dt}\right)=relative\;rate\;of\;change\;in\;water\;volume\;lost\;via\;transpiration$
$v_{w}=\left(\frac{dV_{w}}{V_{w}\;dt}\right)=relative\;rate\;of\;change\;in\;water\;volume\;in\;the\;cell$
ε=volumetric elastic modulus of the cell wall
ϕ=relative irreversible extensibility of the cell wall
Π=dimensionless number
Δπ=osmotic pressure difference across the plasma membrane
*L* (*Δπ − P*) = *relative volumetric rate of water uptake*
*ϕ* (*P − P_C_*) = *relative volumetric plastic deformation rate of the cell wall*
$\frac{dP}{\varepsilon \;dt}=relative\;volumetric\;elastic\;deformation\;rate\;of\;the\;cell\;wall$

## Appendix A

### How Biophysical Variables Are Obtained Experimentally

The magnitude of each Π parameter in Table 2 can be determined for each walled cell from the magnitude of the biophysical variables (*v*_s_, *v*_sT_, *L*, *P*_C_, *ε*, and *ϕ*) determined for that cell; see Table 1. The biophysical variables used in the dimensionless Π parameters are obtained from pressure probe studies conducted on the growing sporangiophores of *P. blakesleeanus*, the internodes of *C. corallina*, and the cells in the stem tissue of *P. satinis* L. See Table A1 for references for each biophysical variable.

**Table A1:** References for each biophysical variable (*v*_s_, *v*_sT_, *L*, *P*_C_, *ε*, and *ϕ*) determined for the four different cell types; the stage I and stage IV sporangiophores of *P. blakesleeanus*, the algal internode cells of *C. corallina*, and the cells from the stem tissue of *P. satinis* L. The values obtained from in vivo creep and in vivo stress relaxation experiments are designated with C and SR, respectively. The transpiration rate is zero for the stage IV sporangiophores of *P. blakesleeanus* during stress relaxation experiments (stage 4-SR), the submerged algal internode cells of *C. corallina*, and the interior cells from the growing tissue of the stem of *P. satinis* L., so NT (not transpiring) is indicated in the table. The transpiration rates for the stage IV sporangiophores (stage 4-C) were measured, but those for stage I were not, so there is no reference for this variable.

	*P. blakesleeanus* Stage 1-C	*P. blakesleeanus* Stage 4-C	*P. blakesleeanus* Stage 4-SR	*C. corallina* Internode-C	*P. satinis* L. Stem-C, SR
*v*_s_ (h^−1^)	[21]	[6]	[6]	[8,9]	[10]
*L* (h^−1^ MPa^−1^)	[11]	[11]	[11]	[12]	[10]
*P*_C_ (MPa)	[21]	[6]	[6]	[8]	[10]
*v*_sT_ (h^−1^)		[13,22]	NT	NT	NT
*ε* (MPa)	[7]	[7,23]	[7,23]	[8]	[10,24]
*ϕ* (h^−1^ MPa^−1^)	[21]	[6]	[6]	[8]	[10]

## Appendix B

### Calculations of Π_wd_

The Π_wd_ parameter is the magnitude of the ratio of the *net* water uptake rate and the *total* wall deformation rate.

$$\Pi_{\mathrm{wd}}=\left(\frac{\Pi_{\mathrm{wv}}-\Pi_{\mathrm{Tv}}}{\Pi_{\mathrm{pv}}+\Pi_{\mathrm{ev}}}\right)$$

The values for the biophysical variables used in the calculations are obtained from [4,9,10,11,12,13,14,15,16].

Estimates of Π_wd_ for the steady and intact growing sporangiophores (*P. blakesleeanus*) from in vivo creep experiments

$$\Pi_{\mathrm{wd}}\left(\mathrm{stage}\;I-C\right)=\left(\frac{\frac{L\;P_{C}}{v_{s}}-\frac{v_{\mathrm{sT}}}{v_{s}}}{\frac{\phi \;P_{C}}{v_{s}}+\frac{P_{C}}{\varepsilon }}\right)=\left(\frac{\frac{\left(\frac{83.0}{h\;MPa}\right)\;\left(0.4\;MPa\right)}{\frac{0.021}{h}}-\frac{\left(\frac{0.12}{h}\right)}{\frac{0.021}{h}}}{\frac{\left(\frac{0.35}{h\;MPa}\right)\;\left(0.4\;MPa\right)}{\frac{0.021}{h}}+\frac{\left(0.4\;MPa\right)}{\left(68.9\;MPa\right)}}\right)\approx 236.0$$

$$\Pi_{\mathrm{wd}}\left(\mathrm{stage}\;\mathrm{IV}-C\right)=\left(\frac{\frac{L\;P_{C}}{v_{s}}-\frac{v_{\mathrm{sT}}}{v_{s}}}{\frac{\phi \;P_{C}}{v_{s}}+\frac{P_{C}}{\varepsilon }}\right)=\left(\frac{\frac{\left(\frac{23.0}{h\;MPa}\right)\;\left(0.26\;MPa\right)}{\frac{0.068}{h}}-\frac{\left(\frac{0.12}{h}\right)}{\frac{0.068}{h}}}{\frac{\left(\frac{2.0}{h\;MPa}\right)\;\left(0.26\;MPa\right)}{\frac{0.068}{h}}+\frac{\left(0.26\;MPa\right)}{\left(60.9\;MPa\right)}}\right)\approx 11.3$$

Estimates of Π_wd_ for the plucked stage IV sporangiophores (*P. blakesleeanus*) from a stress relaxation experiment

$$\Pi_{\mathrm{wd}}\left(\mathrm{stage}\;\mathrm{IV}-\mathrm{SR}\right)=\left(\frac{\frac{L\;P_{C}}{v_{s}}-\frac{v_{\mathrm{sT}}}{v_{s}}}{\frac{\phi \;P_{C}}{v_{s}}+\frac{P_{C}}{\varepsilon }}\right)=\left(\frac{\frac{\left(\frac{23.0}{h\;MPa}\right)\;\left(0.08\;MPa\right)}{\frac{0.0083}{h}}-0}{\frac{\left(\frac{0.169}{h\;MPa}\right)\;\left(0.08\;MPa\right)}{\frac{0.0083}{h}}+\frac{\left(0.08\;MPa\right)}{\left(60.9\;MPa\right)}}\right)\approx 136.0$$

Estimates of Π_wd_ for the growing excised internodes of *C. corallina* from in vivo creep experiments

$$\Pi_{\mathrm{wd}}\left(\mathrm{internode}-C\right)=\left(\frac{\frac{L\;P_{C}}{v_{s}}-\frac{v_{\mathrm{sT}}}{v_{s}}}{\frac{\phi \;P_{C}}{v_{s}}+\frac{P_{C}}{\varepsilon }}\right)=\left(\frac{\frac{\left(\frac{3.0}{h\;MPa}\right)\;\left(0.3\;MPa\right)}{\frac{0.0039}{h}}-0}{\frac{\left(\frac{0.022}{h\;MPa}\right)\;\left(0.3\;MPa\right)}{\frac{0.0039}{h}}+\frac{\left(0.3\;MPa\right)}{\left(100.0\;MPa\right)}}\right)\approx 136.0$$

Estimates of Π_wd_ for the growing cells in excised pea stems (*P. satinis* L.) from in vivo creep and stress relaxation experiments

$$\Pi_{\mathrm{wd}}\left(\mathrm{pea}\right)=\left(\frac{\frac{L\;P_{C}}{v_{s}}-\frac{v_{\mathrm{sT}}}{v_{s}}}{\frac{\phi \;P_{C}}{v_{s}}+\frac{P_{C}}{\varepsilon }}\right)=\left(\frac{\frac{\left(\frac{2.0}{h\;MPa}\right)\;\left(0.3\;MPa\right)}{\frac{0.0751}{h}}-0}{\frac{\left(\frac{0.25}{h\;MPa}\right)\;\left(0.3\;MPa\right)}{\frac{0.0751}{h}}+\frac{\left(0.3\;MPa\right)}{\left(9.5\;MPa\right)}}\right)\approx 7.8$$
